# Development of a behaviour change intervention to increase care home staff influenza vaccination uptake

**DOI:** 10.1016/j.ijnsa.2025.100387

**Published:** 2025-07-24

**Authors:** Amrish Patel, Sion Scott, Alys Wyn Griffiths, David Wright

**Affiliations:** a School of Economics, University of East Anglia, Norwich, UK; b School of Healthcare, University of Leicester, Leicester, UK; c Division of Neuroscience, University of Sheffield, Sheffield, UK

**Keywords:** Long-term care, Influenza vaccination, Barriers, Facilitators, Intervention development, Behaviour change techniques

## Abstract

**Background:**

To protect care home residents the World Health Organisation recommends that 75 % of care home staff are vaccinated for influenza. In the UK this value is less than 30 %. Previously reported interventions have not been informed by theory and usually only addressed one or two known barriers to uptake. Using behavioural science, we worked with care home staff to develop an intervention which addressed all barriers at both individual and care home level.

**Methods:**

We developed an online questionnaire, derived from the literature, asking staff about barriers and facilitators of flu vaccination. These were prioritised (based on frequency and distinctiveness), then mapped to the Theoretical Domains Framework. Relevant behaviour change techniques were identified. Care home staff selected and designed behaviour change techniques according to affordability, practicability, effectiveness, acceptability, safety and equity (APEASE) via an online questionnaire and workshop.

**Results:**

The prioritised barriers were: lack of time to get vaccinated; insufficient vaccine supplies; vaccination costs; a lack of peers getting vaccinated and beliefs that staff do not need vaccination and that it is ineffective. Six behaviour change techniques were selected and developed into a multi-component intervention: (behaviour change technique 1, Restructure of the physical environment) Free, in care home vaccination clinics for staff; (behaviour change techniques 2–4, Information about health consequences, Salience of consequences and information about others’ approval) information campaign featuring care home staff highlighting non-vaccination risks, (behaviour change techniques 5–6, Information about health consequences and Credible source) information campaign featuring primary care doctor challenging misconceptions.

**Conclusions:**

We developed the first theory and evidence-based intervention specifically to facilitate care home staff flu vaccination uptake. Feasibility and acceptability testing of the intervention followed by definitive trial to assess efficacy in care homes is necessary to inform policy decision-making.

**Contribution of the Paper**

What is already known:

• Care home staff influenza vaccination rates are significantly below World Health Organisation targets in England.
• There are currently no theory-based interventions to increase influenza vaccination rates in care home staff.

What this paper adds:

• A theory-based intervention is co-developed with care home staff by exploring barriers and enablers to vaccine uptake, then selecting and contextualising appropriate behaviour change techniques.
• Six behaviour change techniques were found and operationalised as follows: (1) Restructure the physical environment [Pharmacy run, NHS funded, vaccination clinics in care homes for all staff]; (2–4) Information about health consequences, salience of consequences and information about others’ approval [Videos and information materials on the health risk of staff non-vaccination]; and (5–6) Information about health consequences and credible source [A GP in videos and information materials challenging myths around vaccination.]

Alt-text: Unlabelled box

## 1 Background

Each year, seasonal influenza (flu) causes 400,000 deaths globally (Dattani and Spooner, 2022), creating risk for older residents who live in care homes where flu is a major concern (Matias et al., 2016). Care home staff are one of the biggest vectors in this environment, thus, although staff are less likely to die from flu, vaccinating them is critical to reduce the risk of residents catching and dying from flu (Lansbury et al., 2017; Hayward, 2017; Thomas et al., 2016). Evidence suggests a positive linear relationship between staff flu vaccine uptake and resident health outcomes (Wendelboe et al., 2015), thus increasing staff vaccination rates would provide significant benefits to residents, in terms of reduced hospitalisations and deaths (Lansbury et al., 2017; Hayward, 2017; Thomas et al., 2016).

The World Health Organization (WHO) recommends that at least 75 % of health and social care staff are vaccinated for flu (Assembly, 2003). The figure was reported at only 25 % for social care staff in England (Burki, 2018). For 2020–21, a 34 % flu vaccination rate was reported for care home staff (NHS Capacity Tracker (NHS, 2021)), despite the COVID-19 pandemic. Increasing vaccination rates may reduce illness, hospitalisation and mortality amongst residents (Hayward, 2017), improve staff health (Ng and Lai, 2011), reduce sick days (Pereira et al., 2017), improve care continuity and quality (World Health Organization, 2018) and lower staff cover costs (Franklin and Hochlaf, 2018).

The WHO’s 3C model (Confidence, Complacency and Convenience) of vaccine hesitancy (World Health Organization, 2014) summarises barriers and enablers. Confidence includes whether staff believe that: the vaccine is safe and effective; the system and people delivering the vaccine are competent; and, policymakers motivations can be trusted. Complacency includes whether staff believe that: the risk of contracting flu is high; and, flu will negatively impact them and those they care about. Convenience includes whether: staff can easily physically access the vaccine; it is affordable; the quality of the vaccination service is appropriate and the cultural context supports vaccination. This model aligns well with the barriers to flu vaccine uptake identified in the existing evidence base on healthcare staff (Dini et al., 2018; Lorenc et al., 2017) and care home staff to date (Craig et al., 2008; Skivington et al., 2021; Cane et al., 2012; Cane et al., 2015).

It is important to apply theory to understand the processes of behaviour change (Craig et al., 2008; Skivington et al., 2021). The Theoretical Domains Framework provides an integrative framework of behaviour change theories for developing interventions (Cane et al., 2012). Identifying which of the 14 domains are important for the target behaviour provides the necessary theoretical understanding to develop an intervention (Cane et al., 2012). Each Theoretical Domains Framework domain has been linked to a taxonomy of behaviour change techniques, the “building blocks” of behaviour change interventions (Cane et al., 2015).

**Fig. 1:**
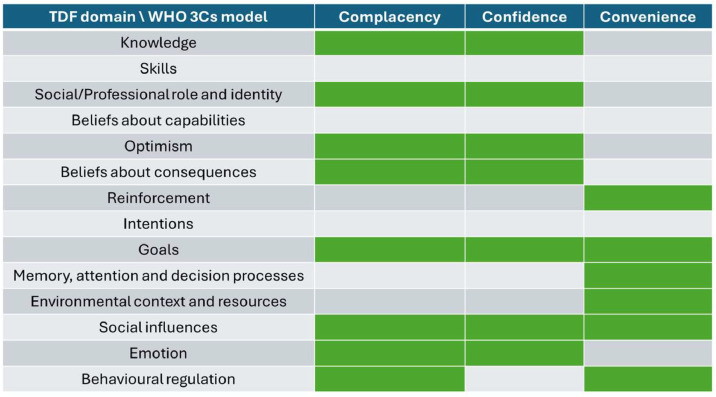
Comparison of Theoretical Domains Framework (TDF) domains and WHO 3Cs model. Note: A shaded cell indicates alignment between the TDF domain and the WHO 3Cs category.

Fig. 1 maps the WHO’s 3C vaccine hesitancy model to the 14 Theoretical Domains Framework domains. It is worth noting that the Theoretical Domains Framework domains (as a more general behaviour change theory framework) covers aspects not in the 3C model (e.g. one’s own skills).

Several policy initiatives in England have attempted to increase flu vaccine uptake in care home staff with limited effect (e.g. NHS funded vaccines; pharmacy vaccinations; flu campaign guidelines (NICE 2020; Public Health England, 2019; England and Improvement, 2019)). These initiatives focus on addressing a single barrier to vaccination at a time rather than implementing a theory-informed intervention designed to overcome all the key barriers and utilise all enablers within care home settings.

The current study is the first to use behaviour change theory to design an intervention that overcomes all the key barriers and utilises all enablers specifically for care home staff flu vaccination uptake. Unlike much of the existing literature on vaccination programme development, the target recipients, care home staff, actively designed the intervention. Our approach provides a useful model community involvement in intervention development for other vaccination programmes or care home interventions.

## 2 Methods

### 2.1 Design

The study had two phases: (1) An online survey was completed by staff working in care homes to prioritise barriers and enablers to flu vaccination requiring addressing by an intervention, (2.1) A second online survey was completed by care home staff to inform the selection of behaviour change techniques behaviour change techniques to address the prioritised barriers, and (2.2) A Nominal Group Technique stakeholder consensus workshop was conducted to finalise and characterise behaviour change techniques. Ethical approval was obtained from the ethics committee at the University of East Anglia.

### 2.2 Participants & procedure

#### 2.2.1 Phase 1: online survey to prioritise barriers to flu vaccination

##### 2.2.1.1 Data collection

Staff who worked in care homes or social care organisations across the UK were eligible to participate. The online survey was distributed via social care organisations (e.g. Care England and National Care Forum), care home social media groups (e.g. on Facebook and LinkedIn), care home staff recruitment companies and direct emails to care homes in July 2020.

The survey asked staff whether they: were vaccinated for flu in 2019–2020; usually get vaccinated for flu; and, planned to be vaccinated in 2020–21. Those reporting that they did not usually get vaccinated for flu were asked to state why by selecting amongst common barriers found in the literature (Kenny et al., 2020; Halpin and Reid, 2019; Shroufi et al., 2009; Alsaif et al., 2023) or clicking “other” and giving their own reasons. Respondents were able to elaborate on any of their responses or share their views on barriers and enablers to flu vaccination in care home staff via a free-text box at the end of the survey. Sociodemographic characteristics were also collected. (See Supplementary File 1 for survey).

##### 2.2.1.2 Data analysis

The research team first coded all free-text responses to produce a complete list of barriers and enablers. Combining the response of the two questions highlighted above, the frequency of each barrier and enabler was measured.

To ensure that the number of barriers and enablers was practical for Phase 2 (where behaviour change techniques are selected and operationalised), the team prioritised the barriers and enablers. Only those with a frequency of at least 2.5 % of the number of responses were eligible to be a priority barrier or enabler, and if a barrier was merely the opposite of an enabler, then only one would be selected.

#### 2.2.2 Phase 2: modified nominal group technique to select and characterise behaviour change techniques

##### 2.2.2.1 Phase 2.1: pre-workshop survey to select behaviour change techniques addressing prioritised barriers

###### 2.2.2.1.1 Data collection

The barriers prioritised in Phase 1 were mapped to the Theoretical Domains Framework (Atkins et al., 2017) and linked to behaviour change techniques identified (Cane et al., 2015) in September 2020.

All identified behaviour change techniques were then initially assessed against the APEASE criteria (affordability, practicability, effectiveness, acceptability, safety and equity; (Michie et al., 2014)) (by AP, SS and AG). Any behaviour change techniques clearly failing in any of the APEASE criteria dimensions were discarded.

SS is a Behavioural Scientist with significant experience in developing behaviour change interventions underpinned by the Theoretical Domains Framework and behaviour change techniques. AP is a behavioural economist with expertise in vaccination uptake and AG is a health psychologist with significant expertise in care homes.

An online survey was then created where each behaviour change technique (and the barrier it was addressing) was described in plain English. Respondents were asked to rate how well the behaviour change technique satisfies each of the APEASE criteria (strongly agree, agree, disagree, strongly disagree). See Supplementary File 2 for survey.

Individuals who had participated in Phase 1 and provided their contact details to take part in further research were invited to take the survey. Additional individuals were also approached via existing researcher networks.

###### 2.2.2.1.2 Data analysis

All behaviour change techniques were classified as “accepted”, “partially accepted”, or “rejected”. A behaviour change technique was accepted if for each of the APEASE criteria, at least 80 % of respondents stated that they agree or strongly agree that it satisfies the criterion (Diamond et al., 2014). A behaviour change technique is “partially accepted” if at least three of the APEASE criteria had 80 % approval. All other behaviour change techniques are rejected.

##### 2.2.2.2 Phase 2.2: nominal group technique workshop to select and characterise behaviour change techniques

###### 2.2.2.2.1 Data collection

Phase 2.1 survey participants were invited to join a two-hour online nominal group technique workshop in October 2020 and received a voucher on workshop completion. The workshop (conducted by AP, SS and AWG) aimed to finalise and characterise behaviour change techniques to be included in the intervention.

In the workshop, participants were presented with the survey responses for each of the partial consensus behaviour change techniques . A Nominal Group Technique cycle was facilitated consisting of: (1) “silent generation” where participants individually thought about how the behaviour change technique does and does not meet the APEASE criteria; (2) “round robin” where participants share their thoughts for or against the behaviour change technique ; (3) “clarification” where discussion is facilitated; (4) “voting” where each participant votes using the APEASE criteria; and finally (5) “discussion” of whether to accept or reject the behaviour change technique. This was done for each behaviour change technique to reach panel consensus to accept or reject (Scott et al., 2021). The voting round and consensus threshold mirrored the online survey APEASE criteria appraisal.

A further Nominal Group Technique cycle was then facilitated per accepted behaviour change technique for stakeholders to characterise them in terms of how they may be operationalised in an intervention to promote uptake of flu vaccination in care home staff, and how they would implement the behaviour change technique within their work setting. A final round of voting was completed before behaviour change technique characterisation statements were then generated, which were refined and validated by the panel.

###### 2.2.2.2.2 Data analysis

Data was analysed live during the workshop in terms of reporting the new voting outcomes and the panel discussing and agreeing the final operationalisation of each accepted behaviour change technique.

## 3 Results

### 3.1 Phase 1: online survey to prioritise barriers to flu vaccination

A total of 409 care home staff respondents participated. While a sample size estimation was not conducted, this sample size permits estimating the proportion of care home staff believing a particular potential barrier is a true barrier within ± 5 percentage points.

Table 1 presents an overview of sample demographics, the sample has more females and British white respondents than demographics for the social care workforce nationally (see Table 1). We found 38 % of respondents stated that they had been vaccinated in the 2019–20 flu season.

**Table 1:** Phase 1 survey participant demographics.

	N (%)
Role	
Activities Co-ordinator	7 (1.7)
Administration	27 (6.6)
(Senior) Care Assistant	204 (49.9)
Manager/Deputy Manager	84 (20.5)
Non-care roles	31 (7.6)
Nurse	37 (9.0)
Organisational level management	8 (2.0)
*Missing*	*11 (2.7)*
Gender	
Female	362 (88.5)
Male	42 (10.3)
*Missing*	*5 (1.2)*
Age	
18–30	68 (16.6)
31–40	70 (17.1)
41–50	86 (21.0)
51–60	133 (32.5)
61+	51 (12.5)
*Missing*	*1 (0.2)*
Ethnicity	
White British	328 (80.2)
Black African/Caribbean	18 (4.40)
Asian/British Asian	19 (4.65)
White European	37 (9.05)
*Missing*	*7 (1.71)*

Note: National demographics for social care staff are: 79 % female; 68 % White; 64 % are 25–54 years old and 27 % are over 55 years old (mean age 44.1 years old) (Skills for Care 2024).

Table 2 lists the barriers and enablers identified, their frequency and whether they were prioritised. Table 3 gives more details of each of the six prioritised barriers, including each barrier being mapped to the WHO 3Cs model (where relevant) and the Theoretical Domains Framework, and illustrative participant responses for each.

**Table 2:** Frequency of barriers and enablers in Phase 1 survey and those prioritised.

		Frequency	Prioritised	Rationale if not prioritised
Barriers				
1	Staff believe that the vaccine is ineffective or causes flu	105	Yes	N/a
2	Staff believe they are fit and healthy, so don’t need vaccination	102	Yes	n/a
3	Staff do not have the time to get to go to a GP/Pharmacy to get vaccinated	45	Yes	n/a
4	Some staff (e.g. agency) have to pay for the vaccine	32	Yes	n/a
5	Insufficient vaccine stock	13	Yes	n/a
6	Staff question why they should get vaccinated when others don’t	11	Yes	n/a
7	Staff are unaware of where to get vaccinated	8	No	Frequency less than 10
8	Staff have needle phobia	3	No	Frequency less than 10
Enablers				
1	Staff believe that they should get vaccinated to protect themselves and residents	49	No	To be utilised when addressing barriers 1 and 2
2	Offering staff vaccinations within the care home	23	No	To be utilised when addressing barriers 3–5
3	Ensuring the vaccine is free for staff	13	No	To be utilised when addressing barrier 4
4	Policies to make flu vaccination mandatory	13	No	Not compliant with current legislation
5	Care home managers being motivated to increase vaccine uptake	9	No	Frequency less than 10
6	Pre-existing conditions implying staff have an additional need to be vaccinated	1	No	Frequency less than 10

**Table 3:** Prioritised barriers to care home staff vaccine uptake and mapping to the Theoretical Domains Framework (TDF) and WHO 3Cs model.

Prioritised barrier	WHO 3Cs model	TDF domain	Illustrative participant response
(1) *Access:* Staff reported lacking time to receive the vaccine	Convenience	Environmental context and resources; Behavioural regulation	“Staff don’t have time to go to their own GP practice when working shifts”
(2) *Vaccine stock*: Pharmacies and GP practices often ran out of vaccine supplies	Convenience	Environmental context and resources	“Access to the vaccine can be patchy, some GPs and chemists don’t have enough and prioritise other groups”
(3) *Cost*: Some care home staff (e.g. agency) have had to pay for their vaccine	Convenience	Environmental context and resources	“Some of my staff find that they can’t get their GP surgery to give vaccines without a cost”
(4) *Perceived lack of need*: Staff perceive themselves as healthy and therefore do not need the vaccine	Complacency	Beliefs about consequences	“I have no underlying health conditions [so don’t need the vaccine]”
(5) *Vaccine beliefs: Some staff believed that the vaccine is either ineffective of causes disease*	Confidence	Beliefs about consequences	“One jab made me ill and put me off having it ever again”
(6) *Peer influence*: Negative influence of anti-vaccination movement. Staff noting colleagues do not have it.	Confidence	Social influences	“I'm not aware of any of my colleagues having the flu jab”

### 3.2 Phase 2: modified nominal group technique to select and characterise behaviour change techniques

#### 3.2.1 Phase 2.1: pre-workshop survey to select behaviour change techniques addressing prioritised barriers

The Theoretical Domains Framework domains were used to identify all relevant behaviour change techniques After the research team had eliminated behaviour change techniques clearly not satisfying the APEASE criteria, 32 potentially appropriate behaviour change techniques remained (see Supplementary File 3).

Twelve participants completed the pre-workshop questionnaire. Table 4 lists the three accepted behaviour change techniques and the eight partially accepted behaviour change techniques along with the barriers they each address (see Supplementary File 3 for full results of all 32 including rationale for their inclusion or exclusion from Phase 2.2).

**Table 4:** Behaviour change techniques (BCTs) accepted or partially accepted in Phase 2.1 survey.

Barrier	BCT (Taxonomy code)	BCT: plain English	Consensus outcome
Staff do not have time to go to the GP/Pharmacy to get vaccinated	Restructuring the physical environment (12.1)	In care home vaccination clinics	Accepted
Staff do not have time to go to the GP/Pharmacy to get vaccinated	Review Goal (1.5)	If vaccination rate remains low, manager to talk to staff to understand why	Accepted
Staff do not have time to go to the GP/Pharmacy to get vaccinated	Problem solving (1.2)	Manager or colleague talks to staff to understand views on vaccination and identify help needed (e.g. pharmacist advice)	Partially accepted
Agency staff have to pay for flu vaccination	Restructuring the physical environment (12.1)	Free flu vaccinations for all care home staff	Accepted
Staff believe they are fit and healthy, so don't need vaccination	Information about health consequences (5.1)	Provide information (e.g. verbal, training, posters, videos) on how vaccination reduces resident illness	Partially accepted
Staff believe they are fit and healthy, so don't need vaccination	Information about others' approval (6.3)	Managers regularly communicate that they strongly approve of staff getting vaccinated. Video of residents explaining that they would like staff vaccinated.	Partially accepted
Insufficient vaccine stock	Adding objects to the environment (12.5)	Care home manager and pharmacist to work together to ear-mark sufficient vaccine stock for all staff	Partially accepted
Staff question why they should get vaccinated when others don't	Information on others' approval (6.3)	Managers communicate that they strongly approve of staff getting vaccinated. Information on how residents and public expect staff vaccinated.	Partially accepted
Staff question why they should get vaccinated when others don't	Framing and reframing (13.2)	Provide information explaining that vaccination is about protecting yourself and your family	Partially accepted
Staff believe that the vaccine is ineffective or causes flu	Information about health consequences (5.1)	Provide information on why mutations means that the vaccine can never be 100 % effective, but even so the effects are large. Explain why it cannot cause flu.	Partially accepted
Staff believe that the vaccine is ineffective or causes flu	Information about social and environmental consequences (5.3)	Provide information about how low vaccination rates and poor infection control can have direct negative effects on the home e.g. forced closure.	Partially accepted

#### 3.2.2 Phase 2.2: Nominal Group Technique workshop to select and characterise behaviour change techniques

Six participants attended the workshop. These consisted of a care home manager, operations manager, registered nurse, quality & training manager, secretary and HR manager, each representing a different care organisation.

**Fig. 2:**
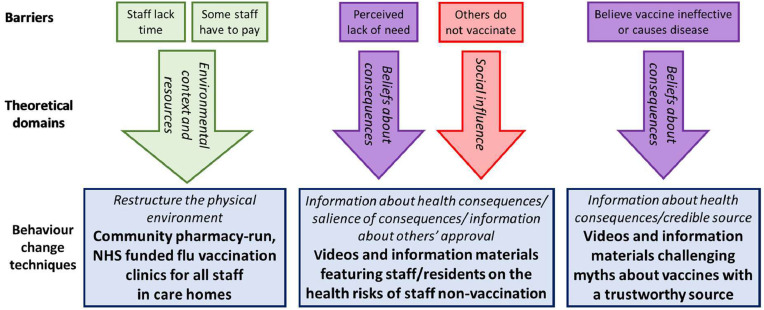
FluCare intervention to increase care home staff flu vaccination rate.

It resulted in six operationalised behaviour change techniques to be included in the intervention (see Fig. 2). behaviour change techniques Taxonomy codes (Michie et al., 2013) follow each behaviour change technique in parentheses.

Behaviour change technique i: Restructure of the physical environment (12.1)

A community pharmacy to offer NHS funded flu vaccination clinics to all staff (inc. agency) in homes. Stakeholders identified that clinics should be run by the pharmacy currently supplying the home’s resident medication to leverage the existing trusted relationship and that several clinics would have to be run at convenient times to account for shift-work and maximise access.

Behaviour change techniques ii-iv: Information about health consequences (5.1), salience of consequences (5.2) and information about others’ approval (6.3) (operationalised together)

Information on the health risks of low staff vaccine uptake featuring staff and residents. An engaging 5–10 min video will be developed, featuring residents and vulnerable staff (older and younger) discussing serious health risks to them arising from poor staff vaccine uptake and how vaccination protects everyone. Videos will be integrated into existing staff processes (e.g. handovers, inductions, staff apps) to ensure engagement. Posters and other information materials will reinforce the main images/messages.

Behaviour change techniques v-vi: Information about health consequences (5.1) and credible source (9.1) (operationalised together)

Information from a trustworthy source such as a General Practitioner, challenging the myths about vaccines. Stakeholders identified a similar format (i.e. short video supported by information materials) and developed some of the myths to be challenged. These included that the vaccine causes flu and is dangerous to pregnant women.

## 4 Discussion

This study identified six main barriers to vaccination uptake and 31 potential behaviour change techniques, which were then refined to six for inclusion in the intervention. All of the main barriers identified in Study 1 map directly onto the WHO 3Cs model of vaccine hesitancy (World Health Organization 2014).

Given our sample size could estimate whether a barrier was a true barrier within ± 5 percentage points, we note that four out of six of our prioritised barriers remain prioritised allowing for this error (Insufficient vaccine stock and staff questioning why they should be vaccinated when others are not, would not meet the 2.5 % prioritisation threshold if we overestimated by 5 % points). Given the liberal prioritisation threshold, all barriers and enablers not prioritised would become prioritised if we had underestimated by 5 % points.

Care home staff identified and evaluated behaviour change techniques for interventions within their workplace setting. This is a key strength of our study, valuing the experience of those working in the setting, rather than those with expertise in behavioural science. This highlights the value of including experts by experience within research, when designing, developing and evaluating potential interventions.

While the proposed intervention targets staff level behaviour change, it is widely recognised that for staff to undertake a behaviour, they must feel it aligns with their organisation’s priorities (Michie et al., 2011). Employer encouragement is a known enabler for staff vaccination (NICE 2020; Shroufi et al., 2009). Care homes receive staff flu campaign guidance (NHS (England and Improvement, 2019); PHE (Public Health England, 2019)) based on a NICE evidence review (NICE, 2020), to facilitate staff vaccination. Therefore, any intervention aiming to increase vaccination rates must consider organisational-level strategies, such as regular monitoring of care homes and feedback on their uptake performance relative to other care homes, or financial incentives for care homes with good staff vaccination rates. Evidence suggests that incentivisation, monitoring and feedback facilitate organisational-level support for behaviour change (e.g. CQUIN financial incentives increasing NHS healthcare staff flu vaccine uptake (Mounier-Jack et al., 2020)).

The main limitation with our study is that all participants worked in senior roles within their organisations (such individuals were more likely to put themselves forward in response to recruitment emails and adverts). It would have been beneficial to have a wider range of staff roles (e.g. care assistants), who may have different views to more senior staff. Furthermore, individuals self-selected their participation, therefore those with strong views on vaccination may have been more likely to participate. Replications of this study with a more diverse set of respondents would be useful to validate the results. It would be important to collect data on the location of respondents as regional heterogeneity is likely.

In line with Medical Research Council (MRC) guidance (Skivington et al., 2021) the next step is to test the intervention for acceptability and practicability with a feasibility study (Patel et al., 2022; Wright et al., 2024). Once this has been revised, its effectiveness and cost-effectiveness requires evaluation within a definitive trial (Wright et al., 2025). With a low baseline vaccinate rate, the sample size required to achieve 75 % levels expected by WHO will be relatively small. Whilst many elements of the intervention are relatively inexpensive, the provision of clinics on-site will require sufficient remuneration to incentivise healthcare professionals to relocate to a different environment and the provision of financial incentives to care home owners will represent a novel approach with the UK’s social care system. Estimating cost-effectiveness of the proposed intervention to describe its potential value to the health system will be necessary to optimise the likelihood of eventual adoption as a national model. The definitive trial would represent the first evaluation of a theory informed intervention to address care home staff influenza vaccination rates.

If found effective, the development of a strategy to facilitate adoption and implementation of the intervention should be guided by theory such as the Behaviour Change Wheel’s policy categories (Michie et al., 2011) and in partnership with policy makers and commissioners. More generally, if effective, the intervention could be adapted to deliver other vaccinations for care home staff (e.g. COVID-19).

This study offers several learnings for developing interventions for the care settings. First, the two stage method used (brief online surveys, then online workshops) is useful for engaging with busy care home staff. Second, recruiting non-managerial care home staff is challenging since they spend less time at a computer. Holding workshops in a care home may help with this. Finally, discussion elements of the workshops were very useful in finalising and operationalising behaviour change techniques given the need for interventions to be appropriate for a diverse range of care homes.

## 5 Conclusions

The present study developed an efficient model that can be used for creating theory informed behaviour change interventions in care home settings. By combining a range of behaviour change techniques into our multi-component intervention we have designed a new, holistic way to increase flu vaccination rates, specifically designed for UK care home staff. The intervention now needs testing for acceptability and practicality prior to feasibility testing and a definitive trial to evaluate its effectiveness and cost-effectiveness.

## Funding

UEA Health & Social Care Partners

## CRediT authorship contribution statement

**Amrish Patel:** Project administration, Funding acquisition, Conceptualization, Writing – review & editing, Methodology, Formal analysis. **Sion Scott:** Supervision, Methodology, Conceptualization, Writing – review & editing, Project administration, Funding acquisition. **Alys Wyn Griffiths:** Writing – original draft, Formal analysis, Conceptualization, Writing – review & editing, Project administration, Data curation. **David Wright:** Writing – review & editing, Funding acquisition, Supervision.

## Declaration of competing interest

No competing interests

## Appendix Supplementary materials

Image, application 1

Image, application 2

Image, application 3
