# Supplementary material for: Development of a behaviour change intervention to increase care home staff influenza vaccination uptake
Source: Int J Nurs Stud Adv. 2025 Jul 24;9:100387. doi: 10.1016/j.ijnsa.2025.100387 (PMC12332908; doi:10.1016/j.ijnsa.2025.100387)
Supplement: Supplementary file 1 [file mmc1.pdf]

# Survey of frontline staff in care homes and other social care settings.

Thank you for taking part in this very short survey.

It will typically take TWO-THREE MINUTES to complete. Most questions are multiple choice.

You can remain completely ANONYMOUS if you wish. This survey is for research purposes only.

For a CHANCE TO WIN £50, simply leave your email address (we will contact you only if you win). Your chance of winning is unaffected by your answers.

Thank you for your participation.

* Required

1. This survey is aimed at people who have worked or are working in the UK. *

   ◯ I confirm I have worked or am working in the UK.

2. Were you working last Autumn/Winter (Sept 2019 to March 2020)? *

   Tick yes even if you did not work for the entire period.

   ◯ Yes

   ◯ No

3. What kind of roles did you hold last autumn/winter? *

Tick as many as apply.

- [ ] Care Assistant/Support Worker
- [ ] Senior Care Assistant
- [ ] Healthcare Assistant
- [ ] Nurse
- [ ] Manager/Deputy Manager/Team Leader
- [ ] Caterer
- [ ] Cleaner
- [ ] Administrator/Secretary
- [ ] Other

4. Where did you work? *

Tick as many as apply.

- [ ] Residential care home (older people)
- [ ] Residential care home (learning/physical disability or mental health)
- [ ] Nursing home (older people)
- [ ] Nursing home (learning/physical disability or mental health)
- [ ] Domiciliary care (providing support in peoples' own homes)
- [ ] Supported living/housing or extra care housing
- [ ] Other

5. What were the terms of your employment last autumn/winter? *

Tick as many as apply.

- [ ] Permanent contract
- [ ] Zero hour contract
- [ ] Agency work
- [ ] Other

6. Were you working for the NHS? *

- ◯ Yes
- ◯ No
- ◯ Yes, but I also worked for private sector care
- ◯ Not sure

7. What is your favorite colour? This is an "attention check" question, please click Green.

- ◯ Red
- ◯ Blue
- ◯ Green
- ◯ Other

8. Did you work part time last Autumn/Winter? *

- ◯ Yes
- ◯ No

9. How many hours did you work in an average week?

- ◯ 1-9
- ◯ 10-19
- ◯ 20-29
- ◯ 30+

10. Did you get vaccinated against flu last year? *

- ◯ No
- ◯ Yes
- ◯ Not sure

11. Did you pay for your vaccination (without reimbursement)? *

- ◯ Yes
- ◯ No
- ◯ Not sure

12. Do you usually get vaccinated for flu? *

- ◯ No
- ◯ Yes

13. Why do you typically not get vaccinated against flu? *

Tick as many as apply.

- ☐ I don't think it prevents flu.
- ☐ I'm too busy.
- ☐ It's too expensive.
- ☐ I don't need it.
- ☐ I don't know where to get vaccinated.
- ☐ Other

14. Do you plan to get vaccinated for flu this year? *

- ◯ Maybe
- ◯ Yes
- ◯ No

15. Does your workplace encourage/promote flu vaccination for staff? *

- ◯ Yes
- ◯ No

16. How do they encourage/promote flu staff vaccination? *

17. Age

- 18-30
- 31-40
- 41-50
- 51-60
- 60+
- Prefer not to say

18. Gender

- Woman
- Man
- Non-binary
- Prefer not to say

19. Ethnicity

- British white
- Other white
- Asian/British Asian
- Black/African/Carribean/Black British
- Mixed/Multiple ethnic groups
- Prefer not to say
- Other

20. (Optional) To be entered into the £50 prize draw please write your email address below (we will only contact you if you've won).

This will be stored securely, not passed onto any third-parties and deleted in December 2020 (when the study ends). We will only contact you if you win.

21. (Optional) We would like to hear more about your views on social care work and flu vaccination. If you are willing to receive a call from our research team please leave your name and phone number below.

This will be stored securely, used only to call you once, not passed onto any third-parties and deleted in December 2020 (when the study ends).

22. (Optional) Even if you don't have time for a chat, please express your views about staff vaccinations in social care settings below. We can only make a case for change if we hear your voice.

For more information on flu vaccination please consult https://www.nhs.uk/conditions/vaccinations/flu-influenza-vaccine/ or https://vk.ovg.ox.ac.uk/vk/inactivated-flu-vaccine.

This content is neither created nor endorsed by Microsoft. The data you submit will be sent to the form owner.

Microsoft Forms
