# Supplementary material for: Development of a behaviour change intervention to increase care home staff influenza vaccination uptake
Source: Int J Nurs Stud Adv. 2025 Jul 24;9:100387. doi: 10.1016/j.ijnsa.2025.100387 (PMC12332908; doi:10.1016/j.ijnsa.2025.100387)
Supplement: Supplementary file 2 [file mmc2.pdf]

# FluCare Pre-Workshop Questionnaire

Thank you for participating in this study.

This questionnaire is part of a research programme on how to improve care home staff flu vaccination rates.

Your answers will help design an intervention that we aim to test in actual care homes.

An "intervention" is any change, strategy or process that could be used in care homes to increase staff flu vaccination. Some might come from care home management, some might come from external partners (e.g. a pharmacist or the NHS).

* Required

## Instructions

Thank you for participating in this study.

In this the next six pages you will see six barriers to staff getting vaccinated for flu.

For each barrier we have provided several intervention components for you to consider. Please rate each component according to whether you think it is:

1. Affordable for your care home
2. Practical to deliver as intended
3. Likely to be effective and cost-effective in addressing the barrier
4. Acceptable to staff and residents in your care home
5. Likely to be safe and free of negative effects (e.g. staff getting stressed).
6. Equitable in that it is unlikely to increase disparities between groups (e.g. by ethnicity or gender).

(After each intervention component we remind you of the barrier that the intervention is trying to deal with).

When appraising each intervention imagine it is really being implemented in your care home (alongside many other interventions) to give a carefully considered rating. Trust your experience and expertise. If you think an intervention might work in principle, but would not work with your colleagues please give it the appropriate low rating.

1. Name *

## Barrier 1: Staff do not have time to go to the GP or pharmacy to get vaccinated

Please consider the following interventions.

2. Intervention 1 (Restructuring physical environment): GPs or pharmacists to run NHS funded flu vaccination clinics in homes. *

   (Barrier to overcome: Staff do not have time to go to the GP or pharmacy to get vaccinated)

| | Strongly agree | Agree | Disagree | Strongly disagree |
|---|---|---|---|---|
| Affordable in my care home | ◯ | ◯ | ◯ | ◯ |
| Practical to deliver as intended | ◯ | ◯ | ◯ | ◯ |
| Likely to be effective and cost-effective | ◯ | ◯ | ◯ | ◯ |
| Acceptable to staff and residents in my home | ◯ | ◯ | ◯ | ◯ |
| Likely to be safe and free from negative effects | ◯ | ◯ | ◯ | ◯ |
| Unlikely to increase existing inequities (e.g. by gender) | ◯ | ◯ | ◯ | ◯ |

3. Intervention 2 (Restructuring physical environment): Care home manager to provide time for staff to get vaccinated during working hours. *

   (Barrier to overcome: Staff do not have time to go to the GP or pharmacy to get vaccinated)

| | Strongly agree | Agree | Disagree | Strongly disagree |
|---|---|---|---|---|
| Affordable in my care home | ◯ | ◯ | ◯ | ◯ |
| Practical to deliver as intended | ◯ | ◯ | ◯ | ◯ |
| Likely to be effective and cost-effective | ◯ | ◯ | ◯ | ◯ |
| Acceptable to staff and residents in my home | ◯ | ◯ | ◯ | ◯ |
| Likely to be safe and free from negative effects | ◯ | ◯ | ◯ | ◯ |
| Unlikely to increase existing inequities (e.g. by gender) | ◯ | ◯ | ◯ | ◯ |

4. Intervention 3 (Action planning): Staff encouraged to plan how the vaccination appointment fits around their work duties and identify when and where to get vaccinated if circumstances change (e.g. work duties take longer than expected). *

(Barrier to overcome: Staff do not have time to go to the GP or pharmacy to get vaccinated)

| | Strongly agree | Agree | Disagree | Strongly disagree |
|---|---|---|---|---|
| Affordable in my care home | ○ | ○ | ○ | ○ |
| Practical to deliver as intended | ○ | ○ | ○ | ○ |
| Likely to be effective and cost-effective | ○ | ○ | ○ | ○ |
| Acceptable to staff and residents in my home | ○ | ○ | ○ | ○ |
| Likely to be safe and free from negative effects | ○ | ○ | ○ | ○ |
| Unlikely to increase existing inequities (e.g. by gender) | ○ | ○ | ○ | ○ |

5. Intervention 4 (Goal setting): Care home manager to set a goal of getting a 100% vaccination rate in the home. *

(Barrier to overcome: Staff do not have time to go to the GP or pharmacy to get vaccinated)

| | Strongly agree | Agree | Disagree | Strongly disagree |
|---|---|---|---|---|
| Affordable in my care home | ○ | ○ | ○ | ○ |
| Practical to deliver as intended | ○ | ○ | ○ | ○ |
| Likely to be effective and cost-effective | ○ | ○ | ○ | ○ |
| Acceptable to staff and residents in my home | ○ | ○ | ○ | ○ |
| Likely to be safe and free from negative effects | ○ | ○ | ○ | ○ |
| Unlikely to increase existing inequities (e.g. by gender) | ○ | ○ | ○ | ○ |

6. Intervention 5 (Review goal): If vaccination rates remain low, care home manager to discuss with staff to understand why *

(Barrier to overcome: Staff do not have time to go to the GP or pharmacy to get vaccinated)

| | Strongly agree | Agree | Disagree | Strongly disagree |
|---|---|---|---|---|
| Affordable in my care home | ○ | ○ | ○ | ○ |
| Practical to deliver as intended | ○ | ○ | ○ | ○ |
| Likely to be effective and cost-effective | ○ | ○ | ○ | ○ |
| Acceptable to staff and residents in my home | ○ | ○ | ○ | ○ |
| Likely to be safe and free from negative effects | ○ | ○ | ○ | ○ |
| Unlikely to increase existing inequities (e.g. by gender) | ○ | ○ | ○ | ○ |

7. Intervention 6 (Problem solving): Manager or colleague has a conversation with staff member to discuss views on vaccination and identify any advice or help needed (e.g. from a pharmacist). *

(Barrier to overcome: Staff do not have time to go to the GP or pharmacy to get vaccinated)

| | Strongly agree | Agree | Disagree | Strongly disagree |
|---|---|---|---|---|
| Affordable in my care home | ○ | ○ | ○ | ○ |
| Practical to deliver as intended | ○ | ○ | ○ | ○ |
| Likely to be effective and cost-effective | ○ | ○ | ○ | ○ |
| Acceptable to staff and residents in my home | ○ | ○ | ○ | ○ |
| Likely to be safe and free from negative effects | ○ | ○ | ○ | ○ |
| Unlikely to increase existing inequities (e.g. by gender) | ○ | ○ | ○ | ○ |

8. Intervention 7 (Monitoring by others without feedback): Manager or external organisation (e.g. NHS or CQC) monitors how many staff get vaccinated. *

(Barrier to overcome: Staff do not have time to go to the GP or pharmacy to get vaccinated)

| | Strongly agree | Agree | Disagree | Strongly disagree |
|---|---|---|---|---|
| Affordable in my care home | ◯ | ◯ | ◯ | ◯ |
| Practical to deliver as intended | ◯ | ◯ | ◯ | ◯ |
| Likely to be effective and cost-effective | ◯ | ◯ | ◯ | ◯ |
| Acceptable to staff and residents in my home | ◯ | ◯ | ◯ | ◯ |
| Likely to be safe and free from negative effects | ◯ | ◯ | ◯ | ◯ |
| Unlikely to increase existing inequities (e.g. by gender) | ◯ | ◯ | ◯ | ◯ |

## Barrier 2: Agency staff are not entitled to a free NHS flu vaccination

Please consider the following intervention.

9. Intervention 1 (Restructuring physical environment): Introduce free NHS funded vaccinations for all care home staff (including agency staff). *

(Barrier to overcome: Agency staff are not entitled to a free NHS flu vaccination)

| | Strongly agree | Agree | Disagree | Strongly disagree |
|---|---|---|---|---|
| Affordable in my care home | ◯ | ◯ | ◯ | ◯ |
| Practical to deliver as intended | ◯ | ◯ | ◯ | ◯ |
| Likely to be effective and cost-effective | ◯ | ◯ | ◯ | ◯ |
| Acceptable to staff and residents in my home | ◯ | ◯ | ◯ | ◯ |
| Likely to be safe and free from negative effects | ◯ | ◯ | ◯ | ◯ |
| Unlikely to increase existing inequities (e.g. by gender) | ◯ | ◯ | ◯ | ◯ |

## Barrier 3: Staff believe they are fit and healthy so do not need the flu vaccination

Please consider the following interventions.

10. Intervention 1 (Information about health consequences/emphasizing consequences): Provide information (e.g. verbal, training, posters or videos) on how staff vaccination has been proven to reduce resident illness, whereas non-vaccination can lead to serious illness. *

(Barrier to overcome: Staff believe they are fit and healthy so do not need the flu vaccination)

| | Strongly agree | Agree | Disagree | Strongly disagree |
|---|---|---|---|---|
| Affordable in my care home | ◯ | ◯ | ◯ | ◯ |
| Practical to deliver as intended | ◯ | ◯ | ◯ | ◯ |
| Likely to be effective and cost-effective | ◯ | ◯ | ◯ | ◯ |
| Acceptable to staff and residents in my home | ◯ | ◯ | ◯ | ◯ |
| Likely to be safe and free from negative effects | ◯ | ◯ | ◯ | ◯ |
| Unlikely to increase existing inequities (e.g. by gender) | ◯ | ◯ | ◯ | ◯ |

11. Intervention 2 (Information about social and environment consequences): Provide information (e.g. verbal, training, posters or videos) explaining: that low vaccination rates and poor infection control have direct negative effects on homes e.g. forced home closures. Homes with high vaccination rates and good infection control do not face such pressures. *

(Barrier to overcome: Staff believe they are fit and healthy so do not need the flu vaccination)

| | Strongly agree | Agree | Disagree | Strongly disagree |
|---|---|---|---|---|
| Affordable in my care home | ○ | ○ | ○ | ○ |
| Practical to deliver as intended | ○ | ○ | ○ | ○ |
| Likely to be effective and cost-effective | ○ | ○ | ○ | ○ |
| Acceptable to staff and residents in my home | ○ | ○ | ○ | ○ |
| Likely to be safe and free from negative effects | ○ | ○ | ○ | ○ |
| Unlikely to increase existing inequities (e.g. by gender) | ○ | ○ | ○ | ○ |

12. Intervention 3 (Information about emotional consequences): Provide information (e.g. verbal, training, posters or videos) on how getting vaccinated makes you feel like you are really looking after your residents. *

(Barrier to overcome: Staff believe they are fit and healthy so do not need the flu vaccination)

| | Strongly agree | Agree | Disagree | Strongly disagree |
|---|---|---|---|---|
| Affordable in my care home | ○ | ○ | ○ | ○ |
| Practical to deliver as intended | ○ | ○ | ○ | ○ |
| Likely to be effective and cost-effective | ○ | ○ | ○ | ○ |
| Acceptable to staff and residents in my home | ○ | ○ | ○ | ○ |
| Likely to be safe and free from negative effects | ○ | ○ | ○ | ○ |
| Unlikely to increase existing inequities (e.g. by gender) | ○ | ○ | ○ | ○ |

13. Intervention 4 (Social support): A manager or colleague is given the responsibility of encouraging vaccination. *

(Barrier to overcome: Staff believe they are fit and healthy so do not need the flu vaccination)

| | Strongly agree | Agree | Disagree | Strongly disagree |
|---|---|---|---|---|
| Affordable in my care home | ○ | ○ | ○ | ○ |
| Practical to deliver as intended | ○ | ○ | ○ | ○ |
| Likely to be effective and cost-effective | ○ | ○ | ○ | ○ |
| Acceptable to staff and residents in my home | ○ | ○ | ○ | ○ |
| Likely to be safe and free from negative effects | ○ | ○ | ○ | ○ |
| Unlikely to increase existing inequities (e.g. by gender) | ○ | ○ | ○ | ○ |

14. Intervention 5 (Social comparison): Inform staff of the vaccination rates in their home or vaccination rates in local homes. *

(Barrier to overcome: Staff believe they are fit and healthy so do not need the flu vaccination)

| | Strongly agree | Agree | Disagree | Strongly disagree |
|---|---|---|---|---|
| Affordable in my care home | ○ | ○ | ○ | ○ |
| Practical to deliver as intended | ○ | ○ | ○ | ○ |
| Likely to be effective and cost-effective | ○ | ○ | ○ | ○ |
| Acceptable to staff and residents in my home | ○ | ○ | ○ | ○ |
| Likely to be safe and free from negative effects | ○ | ○ | ○ | ○ |
| Unlikely to increase existing inequities (e.g. by gender) | ○ | ○ | ○ | ○ |

15. Intervention 6 (Social comparison): Display posters of care home staff getting vaccinated. *

(Barrier to overcome: Staff believe they are fit and healthy so do not need the flu vaccination)

| | Strongly agree | Agree | Disagree | Strongly disagree |
|---|---|---|---|---|
| Affordable in my care home | ◯ | ◯ | ◯ | ◯ |
| Practical to deliver as intended | ◯ | ◯ | ◯ | ◯ |
| Likely to be effective and cost-effective | ◯ | ◯ | ◯ | ◯ |
| Acceptable to staff and residents in my home | ◯ | ◯ | ◯ | ◯ |
| Likely to be safe and free from negative effects | ◯ | ◯ | ◯ | ◯ |
| Unlikely to increase existing inequities (e.g. by gender) | ◯ | ◯ | ◯ | ◯ |

16. Intervention 7 (Information about others' approval): Managers regularly communicate that they strongly approve of staff getting vaccinated. A video of residents explaining how they would like staff to be vaccinated. *

(Barrier to overcome: Staff believe they are fit and healthy so do not need the flu vaccination)

| | Strongly agree | Agree | Disagree | Strongly disagree |
|---|---|---|---|---|
| Affordable in my care home | ◯ | ◯ | ◯ | ◯ |
| Practical to deliver as intended | ◯ | ◯ | ◯ | ◯ |
| Likely to be effective and cost-effective | ◯ | ◯ | ◯ | ◯ |
| Acceptable to staff and residents in my home | ◯ | ◯ | ◯ | ◯ |
| Likely to be safe and free from negative effects | ◯ | ◯ | ◯ | ◯ |
| Unlikely to increase existing inequities (e.g. by gender) | ◯ | ◯ | ◯ | ◯ |

## Barrier 4: Not enough vaccine stock

Please consider the following intervention.

17. Intervention 1 (Adding objects to the environment): Care home manager and pharmacist or GP work together to pre-order and ear-mark sufficient NHS funded vaccine stock for staff to get vaccinated. *

(Barrier to overcome: Not enough vaccine stock)

| | Strongly agree | Agree | Disagree | Strongly disagree |
|---|---|---|---|---|
| Affordable in my care home | ○ | ○ | ○ | ○ |
| Practical to deliver as intended | ○ | ○ | ○ | ○ |
| Likely to be effective and cost-effective | ○ | ○ | ○ | ○ |
| Acceptable to staff and residents in my home | ○ | ○ | ○ | ○ |
| Likely to be safe and free from negative effects | ○ | ○ | ○ | ○ |
| Unlikely to increase existing inequities (e.g. by gender) | ○ | ○ | ○ | ○ |

## Barrier 5: Staff question why they should get vaccinated when others do not.

Please consider the following interventions.

18. Intervention 1 (Social support): Manager praises staff for getting vaccinated. *

(Barrier to overcome: Staff question why they should get vaccinated when other do not)

| | Strongly agree | Agree | Disagree | Strongly disagree |
|---|---|---|---|---|
| Affordable in my care home | ○ | ○ | ○ | ○ |
| Practical to deliver as intended | ○ | ○ | ○ | ○ |
| Likely to be effective and cost-effective | ○ | ○ | ○ | ○ |
| Acceptable to staff and residents in my home | ○ | ○ | ○ | ○ |
| Likely to be safe and free from negative effects | ○ | ○ | ○ | ○ |
| Unlikely to increase existing inequities (e.g. by gender) | ○ | ○ | ○ | ○ |

19. Intervention 2 (Social support practical): Manager to make time for staff to get vaccinated during work hours or between shifts. *

(Barrier to overcome: Staff question why they should get vaccinated when other do not)

| | Strongly agree | Agree | Disagree | Strongly disagree |
|---|---|---|---|---|
| Affordable in my care home | ○ | ○ | ○ | ○ |
| Practical to deliver as intended | ○ | ○ | ○ | ○ |
| Likely to be effective and cost-effective | ○ | ○ | ○ | ○ |
| Acceptable to staff and residents in my home | ○ | ○ | ○ | ○ |
| Likely to be safe and free from negative effects | ○ | ○ | ○ | ○ |
| Unlikely to increase existing inequities (e.g. by gender) | ○ | ○ | ○ | ○ |

20. Intervention 3 (Demonstration of behaviour): Provide a video showing what happens when you get vaccinated. *

(Barrier to overcome: Staff question why they should get vaccinated when other do not)

| | Strongly agree | Agree | Disagree | Strongly disagree |
|---|---|---|---|---|
| Affordable in my care home | ◯ | ◯ | ◯ | ◯ |
| Practical to deliver as intended | ◯ | ◯ | ◯ | ◯ |
| Likely to be effective and cost-effective | ◯ | ◯ | ◯ | ◯ |
| Acceptable to staff and residents in my home | ◯ | ◯ | ◯ | ◯ |
| Likely to be safe and free from negative effects | ◯ | ◯ | ◯ | ◯ |
| Unlikely to increase existing inequities (e.g. by gender) | ◯ | ◯ | ◯ | ◯ |

21. Intervention 4 (Information of others' approval): Managers regularly communicate that they strongly approve of staff getting vaccinated. Information provided about how residents and the general public expect staff vaccination. *

(Barrier to overcome: Staff question why they should get vaccinated when other do not)

| | Strongly agree | Agree | Disagree | Strongly disagree |
|---|---|---|---|---|
| Affordable in my care home | ◯ | ◯ | ◯ | ◯ |
| Practical to deliver as intended | ◯ | ◯ | ◯ | ◯ |
| Likely to be effective and cost-effective | ◯ | ◯ | ◯ | ◯ |
| Acceptable to staff and residents in my home | ◯ | ◯ | ◯ | ◯ |
| Likely to be safe and free from negative effects | ◯ | ◯ | ◯ | ◯ |
| Unlikely to increase existing inequities (e.g. by gender) | ◯ | ◯ | ◯ | ◯ |

22. Intervention 5 (Credible source): Present a speech/video of a senior respectable person or celebrity carer getting vaccinated and explaining its importance. *

(Barrier to overcome: Staff question why they should get vaccinated when other do not)

| | Strongly agree | Agree | Disagree | Strongly disagree |
|---|---|---|---|---|
| Affordable in my care home | ○ | ○ | ○ | ○ |
| Practical to deliver as intended | ○ | ○ | ○ | ○ |
| Likely to be effective and cost-effective | ○ | ○ | ○ | ○ |
| Acceptable to staff and residents in my home | ○ | ○ | ○ | ○ |
| Likely to be safe and free from negative effects | ○ | ○ | ○ | ○ |
| Unlikely to increase existing inequities (e.g. by gender) | ○ | ○ | ○ | ○ |

23. Intervention 6 (Pros and cons): Ask the staff member to write a list of pros and cons of vaccination. *

(Barrier to overcome: Staff question why they should get vaccinated when other do not)

| | Strongly agree | Agree | Disagree | Strongly disagree |
|---|---|---|---|---|
| Affordable in my care home | ○ | ○ | ○ | ○ |
| Practical to deliver as intended | ○ | ○ | ○ | ○ |
| Likely to be effective and cost-effective | ○ | ○ | ○ | ○ |
| Acceptable to staff and residents in my home | ○ | ○ | ○ | ○ |
| Likely to be safe and free from negative effects | ○ | ○ | ○ | ○ |
| Unlikely to increase existing inequities (e.g. by gender) | ○ | ○ | ○ | ○ |

24. Intervention 7 (Identification of self as role model/Identity associated with changed behaviour): Inform staff that they may be a role model for others if they get vaccinated and emphasise their identity as a carer who puts residents first. *

(Barrier to overcome: Staff question why they should get vaccinated when other do not)

| | Strongly agree | Agree | Disagree | Strongly disagree |
|---|---|---|---|---|
| Affordable in my care home | ◯ | ◯ | ◯ | ◯ |
| Practical to deliver as intended | ◯ | ◯ | ◯ | ◯ |
| Likely to be effective and cost-effective | ◯ | ◯ | ◯ | ◯ |
| Acceptable to staff and residents in my home | ◯ | ◯ | ◯ | ◯ |
| Likely to be safe and free from negative effects | ◯ | ◯ | ◯ | ◯ |
| Unlikely to increase existing inequities (e.g. by gender) | ◯ | ◯ | ◯ | ◯ |

25. Intervention 8 (Framing/reframing): Provide information (e.g. posters) explaining that vaccination is about protecting yourself and your family. *

(Barrier to overcome: Staff question why they should get vaccinated when other do not)

| | Strongly agree | Agree | Disagree | Strongly disagree |
|---|---|---|---|---|
| Affordable in my care home | ◯ | ◯ | ◯ | ◯ |
| Practical to deliver as intended | ◯ | ◯ | ◯ | ◯ |
| Likely to be effective and cost-effective | ◯ | ◯ | ◯ | ◯ |
| Acceptable to staff and residents in my home | ◯ | ◯ | ◯ | ◯ |
| Likely to be safe and free from negative effects | ◯ | ◯ | ◯ | ◯ |
| Unlikely to increase existing inequities (e.g. by gender) | ◯ | ◯ | ◯ | ◯ |

26. Intervention 9 (Valued self-identity): Ask staff member to write down their personal strengths as a carer. Where possible, explain that their strengths should mean they vaccinate despite others not doing so. *

(Barrier to overcome: Staff question why they should get vaccinated when other do not)

| | Strongly agree | Agree | Disagree | Strongly disagree |
|---|---|---|---|---|
| Affordable in my care home | ◯ | ◯ | ◯ | ◯ |
| Practical to deliver as intended | ◯ | ◯ | ◯ | ◯ |
| Likely to be effective and cost-effective | ◯ | ◯ | ◯ | ◯ |
| Acceptable to staff and residents in my home | ◯ | ◯ | ◯ | ◯ |
| Likely to be safe and free from negative effects | ◯ | ◯ | ◯ | ◯ |
| Unlikely to increase existing inequities (e.g. by gender) | ◯ | ◯ | ◯ | ◯ |

## Barrier 6: Staff think that the vaccine is ineffective or causes flu.

Please consider the following interventions.

27. Intervention 1 (Information about health consequences): Provide information (e.g. verbal, training, posters or videos) on why mutations means the vaccine can never be 100% effective, but even so the effects are large. Explain why it cannot cause flu. *

    (Barrier to overcome: Staff think that the vaccine is ineffective or causes flu)

| | Strongly agree | Agree | Disagree | Strongly disagree |
|---|---|---|---|---|
| Affordable in my care home | ○ | ○ | ○ | ○ |
| Practical to deliver as intended | ○ | ○ | ○ | ○ |
| Likely to be effective and cost-effective | ○ | ○ | ○ | ○ |
| Acceptable to staff and residents in my home | ○ | ○ | ○ | ○ |
| Likely to be safe and free from negative effects | ○ | ○ | ○ | ○ |
| Unlikely to increase existing inequities (e.g. by gender) | ○ | ○ | ○ | ○ |

28. Intervention 2 (Information about social and environment consequences): Provide information (e.g. verbal, training, posters or videos) explaining: that low vaccination rates and poor infection control have direct negative effects on homes e.g. forced home closures. Homes with high vaccination rates and good infection control do not face such pressures. *

(Barrier to overcome: Staff think that the vaccine is ineffective or causes flu)

| | Strongly agree | Agree | Disagree | Strongly disagree |
|---|---|---|---|---|
| Affordable in my care home | ◯ | ◯ | ◯ | ◯ |
| Practical to deliver as intended | ◯ | ◯ | ◯ | ◯ |
| Likely to be effective and cost-effective | ◯ | ◯ | ◯ | ◯ |
| Acceptable to staff and residents in my home | ◯ | ◯ | ◯ | ◯ |
| Likely to be safe and free from negative effects | ◯ | ◯ | ◯ | ◯ |
| Unlikely to increase existing inequities (e.g. by gender) | ◯ | ◯ | ◯ | ◯ |

29. Intervention 3 (Information about emotional consequences): Provide information (e.g. verbal, training, posters or videos) on how getting vaccinated makes you feel like you are really looking after your residents. *

(Barrier to overcome: Staff think that the vaccine is ineffective or causes flu)

| | Strongly agree | Agree | Disagree | Strongly disagree |
|---|---|---|---|---|
| Affordable in my care home | ◯ | ◯ | ◯ | ◯ |
| Practical to deliver as intended | ◯ | ◯ | ◯ | ◯ |
| Likely to be effective and cost-effective | ◯ | ◯ | ◯ | ◯ |
| Acceptable to staff and residents in my home | ◯ | ◯ | ◯ | ◯ |
| Likely to be safe and free from negative effects | ◯ | ◯ | ◯ | ◯ |
| Unlikely to increase existing inequities (e.g. by gender) | ◯ | ◯ | ◯ | ◯ |

30. Intervention 4 (Social support): Manager or a colleague is given the responsibility of encouraging vaccination. *

(Barrier to overcome: Staff think that the vaccine is ineffective or causes flu)

| | Strongly agree | Agree | Disagree | Strongly disagree |
|---|---|---|---|---|
| Affordable in my care home | ◯ | ◯ | ◯ | ◯ |
| Practical to deliver as intended | ◯ | ◯ | ◯ | ◯ |
| Likely to be effective and cost-effective | ◯ | ◯ | ◯ | ◯ |
| Acceptable to staff and residents in my home | ◯ | ◯ | ◯ | ◯ |
| Likely to be safe and free from negative effects | ◯ | ◯ | ◯ | ◯ |
| Unlikely to increase existing inequities (e.g. by gender) | ◯ | ◯ | ◯ | ◯ |

31. Intervention 5 (Social comparison): Inform staff of the vaccination rates in their home or vaccination rates in local homes. *

(Barrier to overcome: Staff think that the vaccine is ineffective or causes flu)

| | Strongly agree | Agree | Disagree | Strongly disagree |
|---|---|---|---|---|
| Affordable in my care home | ◯ | ◯ | ◯ | ◯ |
| Practical to deliver as intended | ◯ | ◯ | ◯ | ◯ |
| Likely to be effective and cost-effective | ◯ | ◯ | ◯ | ◯ |
| Acceptable to staff and residents in my home | ◯ | ◯ | ◯ | ◯ |
| Likely to be safe and free from negative effects | ◯ | ◯ | ◯ | ◯ |
| Unlikely to increase existing inequities (e.g. by gender) | ◯ | ◯ | ◯ | ◯ |

32. Intervention 6 (Information about others’ approval): Managers regularly communicate that they strongly approve of staff getting vaccinated. Information provided about how residents and the general public expect staff vaccination. *

(Barrier to overcome: Staff think that the vaccine is ineffective or causes flu)

| | Strongly agree | Agree | Disagree | Strongly disagree |
|---|---|---|---|---|
| Affordable in my care home | ◯ | ◯ | ◯ | ◯ |
| Practical to deliver as intended | ◯ | ◯ | ◯ | ◯ |
| Likely to be effective and cost-effective | ◯ | ◯ | ◯ | ◯ |
| Acceptable to staff and residents in my home | ◯ | ◯ | ◯ | ◯ |
| Likely to be safe and free from negative effects | ◯ | ◯ | ◯ | ◯ |
| Unlikely to increase existing inequities (e.g. by gender) | ◯ | ◯ | ◯ | ◯ |
