# Supplementary material for: Development of a behaviour change intervention to increase care home staff influenza vaccination uptake
Source: Int J Nurs Stud Adv. 2025 Jul 24;9:100387. doi: 10.1016/j.ijnsa.2025.100387 (PMC12332908; doi:10.1016/j.ijnsa.2025.100387)
Supplement: Supplementary file 3 [file mmc3.docx]

**Supplementary File 3: BCT Appraisal Using APEASE**

The results of the Phase 2.1 survey appraising 32 BCTs is presented in the table below.

| **Question** | **Barrier** | **TDF Domains** | **BCT** | **BCT: Plain English** |  | **Strongly agree %** | **Agree%** | **Total agreeing %** | **Consensus outcome** |
| --- | --- | --- | --- | --- | --- | --- | --- | --- | --- |
| 2 | Staff do not have time to go to the GP/Pharmacy to get vaccinated | Environmental context and resources; Behavioural Regulation | Restructuring the physical environment | In care home vaccination clinics | Affordability | 41.7 | 58.3 | 100 | Accept |
|  |  |  |  |  | Practicality | 58.3 | 33.3 | 91.6 |  |
|  |  |  |  |  | Effectiveness | 41.7 | 58.3 | 100 |  |
|  |  |  |  |  | Acceptability | 41.7 | 50 | 91.7 |  |
|  |  |  |  |  | Safety | 50 | 41.7 | 91.7 |  |
|  |  |  |  |  | Equity | 33.3 | 58.3 | 91.6 |  |
| 3 | Staff do not have time to go to the GP/Pharmacy to get vaccinated | Environmental context and resources; Behavioural Regulation | Restructuring the physical environment | Time during working hours allocated for vaccination | Affordability | 16.7 | 58.3 | 75 | Reject |
|  |  |  |  |  | Practicality | 8.3 | 50 | 58.3 |  |
|  |  |  |  |  | Effectiveness | 8.3 | 41.7 | 50 |  |
|  |  |  |  |  | Acceptability | 50 | 16.7 | 66.7 |  |
|  |  |  |  |  | Safety | 41.7 | 41.7 | 83.4 |  |
|  |  |  |  |  | Equity | 33.3 | 58.3 | 91.6 |  |
| 4 | Staff do not have time to go to the GP/Pharmacy to get vaccinated | Environmental context and resources; Behavioural Regulation | Action planning | A system to record staff member’s vaccination appointment in work rota or diary including planning how it fits with work. | Affordability | 0 | 58.3 | 58.3 | Reject |
|  |  |  |  |  | Practicality | 16.7 | 33.3 | 50 |  |
|  |  |  |  |  | Effectiveness | 25 | 33.3 | 58.3 |  |
|  |  |  |  |  | Acceptability | 16.7 | 50 | 66.7 |  |
|  |  |  |  |  | Safety | 16.7 | 50 | 66.7 |  |
|  |  |  |  |  | Equity | 8.3 | 58.3 | 66.6 |  |
| 5 | Staff do not have time to go to the GP/Pharmacy to get vaccinated | Environmental context and resources; Behavioural Regulation | Goal setting | Manager sets a 100% goal of vaccination rate in care home | Affordability | 25 | 50 | 75 | Reject |
|  |  |  |  |  | Practicality | 8.3 | 25 | 33.3 |  |
|  |  |  |  |  | Effectiveness | 16.7 | 58.3 | 75 |  |
|  |  |  |  |  | Acceptability | 8.3 | 16.7 | 25 |  |
|  |  |  |  |  | Safety | 16.7 | 50 | 66.7 |  |
|  |  |  |  |  | Equity | 16.7 | 66.7 | 83.4 |  |
| 6 | Staff do not have time to go to the GP/Pharmacy to get vaccinated | Environmental context and resources; Behavioural Regulation | Review Goal | If vaccination rate remains low, manager to talk to staff to understand why | Affordability | 58.3 | 33.3 | 91.6 | Accept |
|  |  |  |  |  | Practicality | 50 | 50 | 100 |  |
|  |  |  |  |  | Effectiveness | 50 | 50 | 100 |  |
|  |  |  |  |  | Acceptability | 41.7 | 58.3 | 100 |  |
|  |  |  |  |  | Safety | 41.7 | 58.3 | 100 |  |
|  |  |  |  |  | Equity | 41.7 | 50 | 91.7 |  |
| 7 | Staff do not have time to go to the GP/Pharmacy to get vaccinated | Environmental context and resources; Behavioural Regulation | Problem solving | Manager or colleague talks to staff to understand views on vaccination and identify help needed (e.g. pharmacist advice) | Affordability | 50 | 33.3 | 83.3 | Partial |
|  |  |  |  |  | Practicality | 41.7 | 50 | 91.7 |  |
|  |  |  |  |  | Effectiveness | 41.7 | 41.7 | 83.4 |  |
|  |  |  |  |  | Acceptability | 33.3 | 41.7 | 75 |  |
|  |  |  |  |  | Safety | 33.3 | 58.3 | 91.6 |  |
|  |  |  |  |  | Equity | 16.7 | 83.3 | 100 |  |
| 8 | Staff do not have time to go to the GP/Pharmacy to get vaccinated | Environmental context and resources; Behavioural Regulation | Monitoring by others without feedback | Manager or extenal organisation (e.g. CQC) monitor vaccination rate | Affordability | 25 | 33.3 | 58.3 | Reject |
|  |  |  |  |  | Practicality | 33.3 | 50 | 83.3 |  |
|  |  |  |  |  | Effectiveness | 25 | 50 | 75 |  |
|  |  |  |  |  | Acceptability | 33.3 | 25 | 58.3 |  |
|  |  |  |  |  | Safety | 33.3 | 33.3 | 66.6 |  |
|  |  |  |  |  | Equity | 25 | 50 | 75 |  |
| 9 | Agency staff have to pay for flu vaccination | Environmental context and resources | Restructuring the physical environment | Free flu vaccinations for all care home staff | Affordability | 75 | 16.7 | 91.7 | Accept |
|  |  |  |  |  | Practicality | 50 | 41.7 | 91.7 |  |
|  |  |  |  |  | Effectiveness | 58.3 | 41.7 | 100 |  |
|  |  |  |  |  | Acceptability | 41.7 | 58.3 | 100 |  |
|  |  |  |  |  | Safety | 41.7 | 58.3 | 100 |  |
|  |  |  |  |  | Equity | 33.3 | 66.7 | 100 |  |
| 10 | Staff believe they are fit and healthy, so don't need vaccination | Beliefs about consequences | Info on health consequences/ emphasising consequences | Provide information (e.g. verbal, training, posters, videos) on how vaccination reduces resident illness | Affordability | 58.3 | 25 | 83.3 | Partial |
|  |  |  |  |  | Practicality | 33.3 | 58.3 | 91.6 |  |
|  |  |  |  |  | Effectiveness | 33.3 | 25 | 58.3 |  |
|  |  |  |  |  | Acceptability | 33.3 | 50 | 83.3 |  |
|  |  |  |  |  | Safety | 33.3 | 50 | 83.3 |  |
|  |  |  |  |  | Equity | 41.7 | 25 | 66.7 |  |
| 11 | Staff believe they are fit and healthy, so don't need vaccination | Beliefs about consequences | Information on social and evironmental consequences | Provide information on how low vaccination rates and poor infection control have direct negative effects on homes (e.g. formed home closures). | Affordability | 33.3 | 41.7 | 75 | Reject |
|  |  |  |  |  | Practicality | 25 | 41.7 | 66.7 |  |
|  |  |  |  |  | Effectiveness | 33.3 | 8.3 | 41.6 |  |
|  |  |  |  |  | Acceptability | 25 | 41.7 | 66.7 |  |
|  |  |  |  |  | Safety | 16.7 | 58.3 | 75 |  |
|  |  |  |  |  | Equity | 25 | 58.3 | 83.3 |  |
| 12 | Staff believe they are fit and healthy, so don't need vaccination | Beliefs about consequences | Information on emptional consequences | Provide information on how getting vaccinated makes you really feel like you're looking after your residents. | Affordability | 41.7 | 33.3 | 75 | Reject |
|  |  |  |  |  | Practicality | 25 | 50 | 75 |  |
|  |  |  |  |  | Effectiveness | 33.3 | 33.3 | 66.6 |  |
|  |  |  |  |  | Acceptability | 25 | 66.7 | 91.7 |  |
|  |  |  |  |  | Safety | 33 | 50 | 83 |  |
|  |  |  |  |  | Equity | 33.3 | 58.3 | 91.6 |  |
| 13 | Staff believe they are fit and healthy, so don't need vaccination | Beliefs about consequences | Social support | A manager or colleague is given responsibility for encouraging vaccination | Affordability | 41.7 | 50 | 91.7 | Reject |
|  |  |  |  |  | Practicality | 33.3 | 41.7 | 75 |  |
|  |  |  |  |  | Effectiveness | 33.3 | 33.3 | 66.6 |  |
|  |  |  |  |  | Acceptability | 16.7 | 58.3 | 75 |  |
|  |  |  |  |  | Safety | 25 | 66.7 | 91.7 |  |
|  |  |  |  |  | Equity | 33.3 | 58.3 | 91.6 |  |
| 14 | Staff believe they are fit and healthy, so don't need vaccination | Beliefs about consequences | Social comparison | Inform staff of vaccination rate in their home or that in local homes | Affordability | 41.7 | 25 | 66.7 | Reject |
|  |  |  |  |  | Practicality | 25 | 41.7 | 66.7 |  |
|  |  |  |  |  | Effectiveness | 25 | 41.7 | 66.7 |  |
|  |  |  |  |  | Acceptability | 33.3 | 33.3 | 66.6 |  |
|  |  |  |  |  | Safety | 25 | 50 | 75 |  |
|  |  |  |  |  | Equity | 25 | 50 | 75 |  |
| 15 | Staff believe they are fit and healthy, so don't need vaccination | Beliefs about consequences | Social comparison | Display posters of care home staff getting vaccinated | Affordability | 25 | 50 | 75 | Reject |
|  |  |  |  |  | Practicality | 33.3 | 41.7 | 75 |  |
|  |  |  |  |  | Effectiveness | 41.7 | 16.7 | 58.4 |  |
|  |  |  |  |  | Acceptability | 25 | 41.7 | 66.7 |  |
|  |  |  |  |  | Safety | 33.3 | 41.7 | 75 |  |
|  |  |  |  |  | Equity | 33.3 | 50 | 83.3 |  |
| 16 | Staff believe they are fit and healthy, so don't need vaccination | Beliefs about consequences | Information about others' approval | Managers regularly communicate that they strongly approve of staff getting vaccinated. Video of residents explaining that they would like staff vaccinated. | Affordability | 41.7 | 41.7 | 83.4 | Partial |
|  |  |  |  |  | Practicality | 41.7 | 33.3 | 75 |  |
|  |  |  |  |  | Effectiveness | 41.7 | 25 | 66.7 |  |
|  |  |  |  |  | Acceptability | 25 | 58.3 | 83.3 |  |
|  |  |  |  |  | Safety | 16.7 | 75 | 91.7 |  |
|  |  |  |  |  | Equity | 33 | 58.3 | 91.3 |  |
| 17 | Insufficient vaccine stock | Environmental context and resources | Adding objects to the environment | Care home manager and pharmacist to work together to ear-mark sufficient vaccine stock for all staff | Affordability | 41.7 | 41.7 | 83.4 | Partial |
|  |  |  |  |  | Practicality | 33.3 | 41.7 | 75 |  |
|  |  |  |  |  | Effectiveness | 41.7 | 41.7 | 83.4 |  |
|  |  |  |  |  | Acceptability | 33.3 | 58.3 | 91.6 |  |
|  |  |  |  |  | Safety | 50 | 50 | 100 |  |
|  |  |  |  |  | Equity | 50 | 50 | 100 |  |
| 18 | Staff question why they should get vaccinated when others don't | Social influences | Social support | Manager praises staff for getting vaccinated | Affordability | 41.7 | 41.7 | 83.4 | Reject |
|  |  |  |  |  | Practicality | 41.7 | 41.7 | 83.4 |  |
|  |  |  |  |  | Effectiveness | 25 | 33.3 | 58.3 |  |
|  |  |  |  |  | Acceptability | 16.7 | 50 | 66.7 |  |
|  |  |  |  |  | Safety | 33.3 | 41.7 | 75 |  |
|  |  |  |  |  | Equity | 25 | 50 | 75 |  |
| 19 | Staff question why they should get vaccinated when others don't | Social influences | Social support practical | Manager to make time for staff to get vaccinated during hours or between shifts | Affordability | 58.3 | 8.3 | 66.6 | Reject |
|  |  |  |  |  | Practicality | 41.7 | 16.7 | 58.4 |  |
|  |  |  |  |  | Effectiveness | 41.7 | 25 | 66.7 |  |
|  |  |  |  |  | Acceptability | 33.3 | 41.7 | 75 |  |
|  |  |  |  |  | Safety | 25 | 66.7 | 91.7 |  |
|  |  |  |  |  | Equity | 25 | 58.3 | 83.3 |  |
| 20 | Staff question why they should get vaccinated when others don't | Social influences | Demonstration of behaviour | Provide a video showing what happens when you get vaccinated | Affordability | 33.3 | 33.3 | 66.6 | Reject |
|  |  |  |  |  | Practicality | 25 | 41.7 | 66.7 |  |
|  |  |  |  |  | Effectiveness | 25 | 33.3 | 58.3 |  |
|  |  |  |  |  | Acceptability | 16.7 | 50 | 66.7 |  |
|  |  |  |  |  | Safety | 16.7 | 75 | 91.7 |  |
|  |  |  |  |  | Equity | 16.7 | 75 | 91.7 |  |
| 21 | Staff question why they should get vaccinated when others don't | Social influences | Information on others' approval | Managers communicate that they strongly approve of staff getting vaccinated. Information on how residents and public expect staff vaccinated. | Affordability | 33.3 | 50 | 83.3 | Partial |
|  |  |  |  |  | Practicality | 33.3 | 50 | 83.3 |  |
|  |  |  |  |  | Effectiveness | 33.3 | 33.3 | 66.6 |  |
|  |  |  |  |  | Acceptability | 16.7 | 58.3 | 75 |  |
|  |  |  |  |  | Safety | 16.7 | 58.3 | 75 |  |
|  |  |  |  |  | Equity | 16.7 | 66.7 | 83.4 |  |
| 22 | Staff question why they should get vaccinated when others don't | Social influences | Credible source | Screen video of senior respectable person or celebrity carer getting vaccinated and explaining its importance | Affordability | 25 | 41.7 | 66.7 | Reject |
|  |  |  |  |  | Practicality | 25 | 33.3 | 58.3 |  |
|  |  |  |  |  | Effectiveness | 33.3 | 8.3 | 41.6 |  |
|  |  |  |  |  | Acceptability | 25 | 41.7 | 66.7 |  |
|  |  |  |  |  | Safety | 16.7 | 58.3 | 75 |  |
|  |  |  |  |  | Equity | 16.7 | 66.7 | 83.4 |  |
| 23 | Staff question why they should get vaccinated when others don't | Social influences | Pros and cons | Staff to write a list of pros and cons | Affordability | 25 | 41.7 | 66.7 | Reject |
|  |  |  |  |  | Practicality | 25 | 41.7 | 66.7 |  |
|  |  |  |  |  | Effectiveness | 16.7 | 33.3 | 50 |  |
|  |  |  |  |  | Acceptability | 8.3 | 41.7 | 50 |  |
|  |  |  |  |  | Safety | 8.3 | 83.3 | 91.6 |  |
|  |  |  |  |  | Equity | 16.7 | 75 | 91.7 |  |
| 24 | Staff question why they should get vaccinated when others don't | Social influences | Identification of self as role model/identity associated with changed behaviour | Inform staff that they may be a role model for other staff if they get vaccinated and emphasize their identity as a carer who puts residents first | Affordability | 16.7 | 66.7 | 83.4 | Reject |
|  |  |  |  |  | Practicality | 16.7 | 58.3 | 75 |  |
|  |  |  |  |  | Effectiveness | 16.7 | 33.3 | 50 |  |
|  |  |  |  |  | Acceptability | 16.7 | 41.7 | 58.4 |  |
|  |  |  |  |  | Safety | 16.7 | 50 | 66.7 |  |
|  |  |  |  |  | Equity | 8.3 | 75 | 83.3 |  |
| 25 | Staff question why they should get vaccinated when others don't | Social influences | Framing and reframing | Provide information explaining that vaccination is about protecting yourself and your family | Affordability | 25 | 58.3 | 83.3 | Partial |
|  |  |  |  |  | Practicality | 25 | 58.3 | 83.3 |  |
|  |  |  |  |  | Effectiveness | 33.3 | 16.7 | 50 |  |
|  |  |  |  |  | Acceptability | 16.7 | 58.3 | 75 |  |
|  |  |  |  |  | Safety | 16.7 | 58.3 | 75 |  |
|  |  |  |  |  | Equity | 16.7 | 66.7 | 83.4 |  |
| 26 | Staff question why they should get vaccinated when others don't | Social influences | Valued self-identity | Ask staff to write down their personal strengths as a carer. Explain how these strengths imply vaccination. | Affordability | 41.7 | 33.3 | 75 | Reject |
|  |  |  |  |  | Practicality | 33.3 | 33.3 | 66.6 |  |
|  |  |  |  |  | Effectiveness | 25 | 33.5 | 58.5 |  |
|  |  |  |  |  | Acceptability | 16.7 | 50 | 66.7 |  |
|  |  |  |  |  | Safety | 25 | 50 | 75 |  |
|  |  |  |  |  | Equity | 25 | 50 | 75 |  |
| 27 | Staff believe that the vaccine is ineffective or causes flu | Beliefs about consequences | Information about health consequences | Provide information on why mutations means that the vaccine can never be 100% effective, but even so the effects are large. Explain why it cannot cause flu. | Affordability | 16.7 | 75 | 91.7 | Partial |
|  |  |  |  |  | Practicality | 8.3 | 91.7 | 100 |  |
|  |  |  |  |  | Effectiveness | 25 | 50 | 75 |  |
|  |  |  |  |  | Acceptability | 8.3 | 83.3 | 91.6 |  |
|  |  |  |  |  | Safety | 8.3 | 91.7 | 100 |  |
|  |  |  |  |  | Equity | 0 | 100 | 100 |  |
| 28 | Staff believe that the vaccine is ineffective or causes flu | Beliefs about consequences | Information about social and environmental consequences | Provide information about how low vaccination rates and poor infection control can have direct negative effects on the home e.g. forced closure. | Affordability | 16.7 | 75 | 91.7 | Partial |
|  |  |  |  |  | Practicality | 25 | 58.3 | 83.3 |  |
|  |  |  |  |  | Effectiveness | 25 | 33.3 | 58.3 |  |
|  |  |  |  |  | Acceptability | 8.3 | 66.7 | 75 |  |
|  |  |  |  |  | Safety | 8.3 | 75 | 83.3 |  |
|  |  |  |  |  | Equity | 16.7 | 66.7 | 83.4 |  |
| 29 | Staff believe that the vaccine is ineffective or causes flu | Beliefs about consequences | Information on emotional consequences | Provide information on how getting vaccinated makes you really feel like you're looking after your residents. | Affordability | 25 | 58.3 | 83.3 | Reject |
|  |  |  |  |  | Practicality | 25 | 58.3 | 83.3 |  |
|  |  |  |  |  | Effectiveness | 16.7 | 41.7 | 58.4 |  |
|  |  |  |  |  | Acceptability | 16.7 | 50 | 66.7 |  |
|  |  |  |  |  | Safety | 8.3 | 66.7 | 75 |  |
|  |  |  |  |  | Equity | 8.3 | 66.7 | 75 |  |
| 30 | Staff believe that the vaccine is ineffective or causes flu | Beliefs about consequences | Social support | Manager or colleague given responsibility to encourage vaccination | Affordability | 33.3 | 41.7 | 75 | Reject |
|  |  |  |  |  | Practicality | 33.3 | 33.3 | 66.6 |  |
|  |  |  |  |  | Effectiveness | 16.7 | 50 | 66.7 |  |
|  |  |  |  |  | Acceptability | 16.7 | 50 | 66.7 |  |
|  |  |  |  |  | Safety | 8.3 | 66.7 | 75 |  |
|  |  |  |  |  | Equity | 8.3 | 75 | 83.3 |  |
| 31 | Staff believe that the vaccine is ineffective or causes flu | Beliefs about consequences | Social comparison | Inform staff of the vaccination rate in their home or the local area | Affordability | 25 | 58.3 | 83.3 | Reject |
|  |  |  |  |  | Practicality | 25 | 58.3 | 83.3 |  |
|  |  |  |  |  | Effectiveness | 16.7 | 41.7 | 58.4 |  |
|  |  |  |  |  | Acceptability | 16.7 | 50 | 66.7 |  |
|  |  |  |  |  | Safety | 16.7 | 58.3 | 75 |  |
|  |  |  |  |  | Equity | 8.3 | 75 | 83.3 |  |
| 32 | Staff believe that the vaccine is ineffective or causes flu | Beliefs about consequences | Information about others' approval | Managers communicate that they strongly approve of staff getting vaccinated. Information provided on how public and residents expect staff to be vaccinated. | Affordability | 16.7 | 66.7 | 83.4 | Reject |
|  |  |  |  |  | Practicality | 25 | 50 | 75 |  |
|  |  |  |  |  | Effectiveness | 16.7 | 33.3 | 50 |  |
|  |  |  |  |  | Acceptability | 25 | 41.7 | 66.7 |  |
|  |  |  |  |  | Safety | 8.3 | 58.3 | 66.6 |  |
|  |  |  |  |  | Equity | 8.3 | 66.7 | 75 |  |
